# Variability of Gut Microbiota Across the Life Cycle of *Grapholita molesta* (Lepidoptera: Tortricidae)

**DOI:** 10.3389/fmicb.2020.01366

**Published:** 2020-06-30

**Authors:** Xueli Wang, Shengjie Sun, Xuelin Yang, Jie Cheng, Hongshuang Wei, Zhen Li, J. P. Michaud, Xiaoxia Liu

**Affiliations:** ^1^Department of Entomology and MOA Key Laboratory of Pest Monitoring and Green Management, College of Plant Protection, China Agricultural University, Beijing, China; ^2^Department of Entomology, Agricultural Research Center-Hays, Kansas State University, Hays, KS, United States

**Keywords:** oriental fruit moth, microbiota dynamics, bacterial diversity, 16S rRNA, gut microbiota, symbiosis

## Abstract

*Grapholita molesta*, the oriental fruit moth, is a serious global pest of many Rosaceae fruit trees. Gut microorganisms play important roles in host nutrition, digestion, detoxification, and resistance to pathogens. However, there are few studies on the microbiota of *G. molesta*, particularly during metamorphosis. Here, the diversity of gut microbiota across the holometabolous life cycle of *G. molesta* was investigated comprehensively by Illumina high-throughput sequencing technology. The results showed that the microbiota associated with eggs had a high number of operational taxonomic units (OTUs). OTU and species richness in early-instar larvae (first and second instars) were significantly higher than those in late-instar larvae (third to fifth instars). Species richness increased again in male pupae and adults, apparently during the process of metamorphosis, compared to late-instar larvae. Proteobacteria and Firmicutes were the dominant phyla in the gut and underwent notable changes during metamorphosis. At the genus level, gut microbial community shifts from *Gluconobacter* and *Pantoea* in early-instar larvae to *Enterococcus* and *Enterobacter* in late-instar larvae and to *Serratia* in pupae were apparent, in concert with host developmental changes. Principal coordinate analysis (PCoA) and linear discriminant analysis effect size (LEfSe) analyses confirmed the differences in the structure of gut microbiota across different developmental stages. In addition, sex-dependent bacterial community differences were observed. Microbial interaction network analysis showed different correlations among intestinal microbes at each developmental stage of *G. molesta*, which may result from the different abundance and diversity of gut microbiota at different life stages. Phylogenetic Investigation of Communities by Reconstruction of Unobserved States (PICRUSt) analysis indicated that most functional prediction categories of gut microbiota were related to membrane transport, carbohydrate and amino acid metabolism, and DNA replication and repair. Bacteria isolated by conventional culture-dependent methods belonged to Proteobacteria, Firmicutes, and Actinobacteria, which was consistent with high-throughput sequencing results. In conclusion, exploration of gut bacterial community composition in the gut of *G. molesta* should shed light into deeper understanding about the intricate associations between microbiota and host and might provide clues to the development of novel pest management strategies against fruit borers.

## Introduction

The oriental fruit moth, *Grapholita molesta* (Busck; Lepidoptera: Tortricidae), a major pest of Rosaceae fruit trees, is widely distributed throughout the fruit-growing regions of Asia, Europe, America, Australia, and Africa (Dorn et al., 2003; Bellerose et al., 2007; Myers et al., 2007; Timm et al., 2008; Kirk et al., 2013). In China, it occurs in most of the fruit-growing regions except Tibet and can harm apple, pear, jujube, peach, plum, apricot, hawthorn, and other fruit trees by boring and feeding in twigs and fruits at larval stage, resulting in shoot dieback and fruit shedding (Myers et al., 2006; Cao et al., 2015; Duarte et al., 2015; Tian et al., 2019). *G. molesta* undergoes multiple generations in a year and has the habit of host switching, which cause serious economic losses to the fruit industry every year (Zheng et al., 2015). Intensive use of insecticides in orchard poses risks to fruit quality and environmental contamination and selection pressure on the oriental fruit moth to evolve resistance. Thus, alternative methods for *G. molesta* control are urgently needed.

The gut microbiota, which has been recognized as a virtual “organ,” is integrated into the biological system of the host and indispensable to its health (Backhed et al., 2005; Spor et al., 2011). Numerous studies have shown that gut microbes strongly influence host fitness by playing important roles in host nutrition, digestion, and detoxification and by defending the host against predators, parasites, and pathogens (Dillon et al., 2002; Dorn et al., 2003; Dillon and Dillon, 2004; Myers et al., 2006; Douglas, 2009; Feldhaar, 2011; Spor et al., 2011; Engel and Moran, 2013). For example, *Pantoea agglomerans* and other common gut bacteria of the gregarious locust *Schistocerca gregaria* (Orthoptera: Catantopidae) produce components of aggregation pheromone by breaking down dietary ingredients (Dillon et al., 2002). *Lactobacillus plantarum* is correlated with promoting the systemic growth of *Drosophila melanogaster* (Diptera: Drosophilidae; Storelli et al., 2011). The microorganisms of Sphingomonadaceae are associated with degradation of hexachlorocyclohexane and pyrethroid (Wang et al., 2009; Lal et al., 2010). In some cases, insect gut microbiota is deleterious to host, depending on environmental circumstance or host genotype. The key features of a deleterious phenotype are a high rate of proliferation and high abundance, often accompanied by an expanded distribution within the insect body (Douglas, 2011). For example, translocation of *Enterococcus faecalis* from the gut to the hemocoel of *Manduca sexta* (Lepidoptera: Sphingidae) leads to a switch from commensal to pathogen (Holt et al., 2015). The popcorn strain of *Wolbachia* with high abundance has a negative effect on the longevity of *Drosophila* (McGraw et al., 2002). The effects of gut microbiota on insect fitness traits have now provided new perspectives for the development of new strategies for pest control (Hoffmann et al., 2011; Berasategui et al., 2016; Almeida et al., 2017).

The effects of gut microbes on insects are of relevance to medicine, agriculture, and the field of ecology. Insect–microbe interactions can be critical to the decomposition of plant biomass and carbon cycle (Freitak et al., 2009) and to nitrogen fixation and the nitrogen cycle (Fox-Dobbs et al., 2010), generating various effects on natural and agricultural ecosystems. Thus, understanding the gut microbial community associated with insect hosts is important and necessary for subsequent functional studies. Gut microbial communities of insects are determined by many complex factors, such as environmental habitat, host genetics, diet, sex, and developmental stage (Pinto-Tomas et al., 2011; Campbell et al., 2012; Yun et al., 2014; Chen et al., 2016). With the rapid development of next-generation sequencing technology, a growing number of insect studies have focused on how microbial populations change over host metamorphosis by conventional culture-dependent and culture-independent techniques, such as *Heliconius erato* (Lepidoptera: Nymphalidae; Hammer et al., 2014), *Spodoptera littoralis* (Lepidoptera: Noctuidae; Chen et al., 2016), *Bombyx mori* (Lepidoptera: Bombycidae; Chen et al., 2018), *Chrysoperla sinica* (Neuroptera: Chrysopidae; Zhao et al., 2019), and *Octodonta nipae* (Coleoptera: Chrysomelidae; Ali et al., 2019). Differences in the gut morphology and function in successive life stages may alter microbial diversity (Engel and Moran, 2013). For example, Moll et al. (2001) showed that newly emerged mosquito adults contain few or no bacteria in their guts. Shifts in the microbial community in *B. mori* were apparent between early- and late-instar larvae, in concert with host developmental changes (Chen et al., 2018). As a holometabolous insect, the oriental fruit moth has distinct egg, larval, pupal, and adult stages and undergoes a radical remodeling of the gut and other organs during metamorphosis. To investigate how the microbial community associated with *G. molesta* varies across life stages, the structure of bacterial community across the entire life cycle of *G. molesta* was elucidated for the first time by Illumina sequencing of 16S rRNA genes. Additionally, multiple bacterial populations were cultured from the gut of fifth-instar larvae for subsequent functional studies. Identification of microbiota associated with *G. molesta* across its life cycle represents the first step toward investigating the functional role of microbiota in its host development.

## Materials and Methods

### Insect Rearing and Sample Processing

**FIGURE 1:**
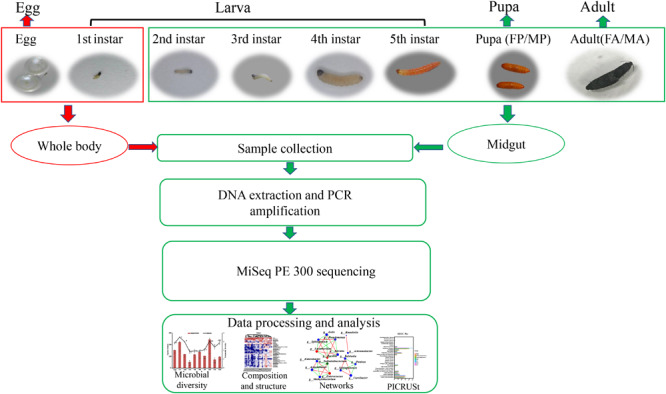
Sample collection of *G. molesta* and 16S rRNA sequencing process. The whole body of eggs and first-instar larvae were used to investigate the internal bacteria because of their tiny size. Abbreviations: 1st = first instar, 2nd = second instar, 3rd = third instar, 4th = fourth instar, 5th = fifth instar, FP = female pupa, MP = male pupa, FA = female adult, and MA = male adult.

*Grapholita molesta* specimens used for this study are derived from colonies that have been reared for more than 6 years under laboratory conditions, at 25 ± 1°C, 70 ± 10% relative humidity (RH), 15:9 (L:D) photoperiod (Cao et al., 2015). Eggs were reared on fresh Fuji apples until the fifth-instar larvae emerged from the apples, at which point, a large piece of gauze was prepared as substrate for pupation. Newly emerged adults were held in a smooth plastic box (250 ml) and fed with 10% honey solution, and the female adults laid their eggs on the smooth plastic box walls. Samples of 3-day-old eggs, larvae (first to fifth instar), 1-day-old pupae (male and female), and 1-day-old adults (male and female) were collected for 16S rRNA sequencing. For sample processing, all insects were rinsed three times in sterile water, surface-sterilized in 70% ethanol for 30 s, and rinsed again in sterile water. Then the whole midgut tissue was dissected from second- to fifth-instar larvae (20, 20, 10, and 10 larvae per replicate, respectively), female and male pupae (15 pupae per replicate), and female and male adults (15 adults per replicate) in a sterile environment. The whole body of eggs (150 eggs per replicate) and first-instar larvae (150 larvae per replicate) were used to investigate the internal bacteria because of their tiny size. This generated a total of 10 treatments (life stages) that were analyzed with three replicates of each. DNA was then extracted from these samples (midgut and whole body) and sequenced by Illumina MiSeq PE300. The specific experimental design and high-throughput sequencing process were depicted schematically in Figure 1.

### DNA Extraction and PCR Amplification

Total DNA of each sample was extracted using the E.Z.N.A.^®^Stool DNA Kit (D4015, Omega, Inc., United States) according to manufacturer’s instructions and stored at −80°C until examination in the PCR by LC-Bio Technology Co., Ltd, Hang Zhou, Zhejiang Province, China. The V3–V4 region of bacterial 16S rRNA gene was amplified using primers 338F (5′-ACTCCTACGGGAGGCAGCAG-3′) and 806R (5′-GGACTACHVGGGTWTCTAAT-3′; Xu et al., 2016). PCR amplification was performed in a total volume of 25 μl reaction mixture containing 25 ng of template DNA, 12.5 μl PCR Premix, 2.5 μl of each primer, and PCR-grade water to adjust the volume. The PCR conditions were as follows: initial denaturation at 98°C for 30 s, followed by 35 cycles of 10 s at 98°C, 30 s at 54°C, 45 s at 72°C, and then a final extension at 72°C for 10 min. PCR products were confirmed with 2% agarose gel electrophoresis. Ultrapure water was used to exclude the possibility of false-positive PCR results as a negative control. The PCR products were purified by AMPure XT beads (Beckman Coulter Genomics, Danvers, MA, United States) and quantified by Qubit (Invitrogen, United States). The amplicon pools were prepared for sequencing, and the size and quantity of the amplicon library were assessed on an Agilent 2100 Bioanalyzer (Agilent, United States) and with the Library Quantification Kit for Illumina (Kapa Biosciences, Woburn, MA, United States), respectively. The libraries were sequenced on an Illumina MiSeq platform with the PE300 model according to the manufacturer’s recommendations provided by LC-Bio.

### High-Throughput Sequencing and Analysis

The raw 16S rRNA gene sequencing reads were demultiplexed and quality-filtered by Trimmomatic (version 0.30) with the parameters (SLIDINGWINDOW: 50: 20 MINLEN: 50; Bolger et al., 2014) and merged by FLASH (version 1.2.11, https://ccb.jhu.edu/software/FLASH/index.shtml) using the parameters (−m 10 −x 0.2 −p 33 −r 300 −f 450 −s 150; Magoc and Salzberg, 2011); the criteria were as follows: (i) the 300-bp reads were truncated at any site receiving an average quality score of <20 over a 50-bp sliding window, and the truncated reads shorter than 50 bp or those containing ambiguous characters were discarded. (ii) Only sequences with overlaps longer than 10 bp were merged according to their overlapping sequence. The maximum mismatch ratio of the overlapping region is 0.2. Reads that could not be assembled were discarded. (iii) Samples were distinguished according to the barcodes and primers, the sequence direction was adjusted, the mismatch allowed for barcode was 0, and the maximum primer mismatch number was 2 (Chen et al., 2018; Liu et al., 2018; Zhao et al., 2019). Operational taxonomic units (OTUs) with 97% similarity cutoff were clustered using UPARSE (version 7.0.1090, http://www.drive5.com/uparse/; Edgar, 2013), and chimeric sequences, chloroplasts, and mitochondria sequences were identified and removed. The taxonomy of each OTU representative sequence was analyzed by an RDP Classifier (version 11.5, http://rdp.cme.msu.edu/) against the 16S rRNA database Silva SSU128 using a confidence threshold of 0.7 (Quast et al., 2013).

To normalize the sequencing depth of each sample, 30,518 sequences per sample were randomly selected for further analysis. The sobs rarefaction curves and species richness and community diversity indices (Chao1 and Shannon) were estimated using the mothur software (version 1.30.2, https://www.mothur.org/wiki/Download_mothur; Schloss et al., 2009), Welch’s *t* test was performed to compare different treatments. One-way analysis of variance (ANOVA) followed by Duncan’s test was performed to assess the significant differences of the number of OTUs at different life stages by SPSS 21.0 software (Callens et al., 2018; Li et al., 2018; Zhang et al., 2018), and *P* ≤ 0.05 was considered statistically significant. A taxonomic heat map was generated to visualize the distribution of multiple OTUs at different life stages using average clustering by the vegan package (R version 3.3.1; Dixon, 2003). Principal coordinate analysis (PCoA) based on the Bray–Curtis similarities index was applied to rank the bacterial communities. An analysis of similarity (ANOSIM) was performed to determine the differences among treatments. Linear discriminant analysis effect size (LEfSe) analysis was performed using the LEfSe tool (version 1.0, http://huttenhower.sph.harvard.edu/galaxy/root/index) to identify specialized bacterial groups present at each life stage. A network analysis across the life cycle of *G. molesta* was conducted with NetworkX software (version 1.11) with an absolute correlation coefficient greater than 0.8 on the free online platform of Majorbio I-Sanger Cloud Platform. Phylogenetic Investigation of Communities by Reconstruction of Unobserved States (PICRUSt) was used to predict the functional profile of bacterial communities at different life stages. Specific principles and methods followed Langille et al. (2013). The metagenome inference step relied on a table of OTUs for each sample with associated Greengenes identifiers. The resultant biom-formatted OTU table was first normalized with respect to inferred 16S rRNA gene copy numbers, and then functional information corresponding to OTUs was obtained by corresponding Greengenes ID. Putative microbiota functions were exported as Kyoto Encyclopedia of Genes and Genomes (KEGG) orthologs, and one-way ANOVA followed by Duncan’s test was used for comparisons of potential function capacities among different treatments. The raw data obtained in this study were deposited in the NCBI short-read archive (SRA) under accession number SRP256116.

### Isolation and Identification of the Culturable Bacteria From Fifth-Instar Larvae

A traditional culture-dependent method was used to isolate culturable bacteria from the midgut of fifth-instar larvae. Stringent procedures were employed so as to process all samples under sterile conditions. Specifically, fifth-instar larvae feeding on Fuji apples were collected to isolate the gut bacteria. Ten healthy larvae were surface disinfected in 75% ethanol followed by thorough rinsing with sterilized distilled water. Then, the larvae were dissected in a sterilized environment, and the midgut was removed and placed in a 200 μl phosphate buffer saline (pH = 7.4). The larval midgut was homogenized and diluted to the appropriate concentration to spread on Luria Bertani (LB) agar plates (tryptone 10 g/L, yeast extract 5 g/L, NaCl 10 g/L, and agar 15 g/L). The plates were incubated at 37°C, and the differentiation of colonies in size, color, and morphology was observed every 24 h. Thereafter, a single representative isolate of each morphotype was transferred to new plates for three to four times purification, and each purified isolate (three to five colonies) was identified by PCR amplification of 16S rRNA gene using universal bacterial forward primer 27F (5′-AGAGTTTGATCCTGGCTCAG-3′) and reverse primer 1492R (5′-TACGGCTACCTTGTTACGACTT-3′; Thakur et al., 2015; Raza et al., 2020). PCR amplification was carried out with the following programs: 94°C for 3 min, 30 cycles at 94°C for 30 s, 55°C for 30 s, 72°C for 30 s, and a final extension at 72°C for 7 min. Amplification products were examined by electrophoresis in 1% agarose gels containing M5 Gelred Plus (Mei5 Biotechnology Co., Ltd., Beijing, China). Subsequently, PCR products were purified by PCR purification kit (TSINGKE) and then sequenced by bidirectional Sanger sequencing and assembled by SeqMan software. The 16S rRNA sequence of each isolate was compared and aligned with cataloged sequences on the NCBI website using ClustalW. A phylogenetic tree was constructed by neighbor-joining analysis on aligned sequences with MEGA7 software (Thakur et al., 2015). The evolutionary distances were computed using the *p*-distance method (Nei and Kumar, 2000), and all positions containing gaps and missing data were eliminated. Accession numbers of sequences included in the alignments were given before the strain name in Supplementary Figure S3. The nucleotide sequences of isolates 1–17 were submitted to the GenBank database with accession numbers MN826158–MN826174.

## Results

### Diversity of Bacterial Communities Across the Life Cycle of *G. molesta*

**FIGURE 2:**
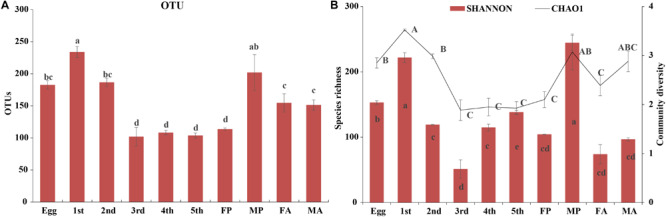
Diversity of bacterial communities across the life cycle of *G. molesta*. **(A)** The number of OTUs across the life cycle of *G. molesta*. **(B)** Species richness (Chao1 index) and community diversity (Shannon index) across the life cycle of *G. molesta*. Abbreviations: Gm = *Grapholita molesta*, 1st = first instar, 2nd = second instar, 3rd = third instar, 4th = fourth instar, 5th = fifth instar, FP = female pupa, MP = male pupa, FA = female adult, and MA = male adult. OTU values bearing different lowercase letters were significantly different (Duncan’s test, *P* < 0.05). Values for Chao1 and Shannon indices bearing different uppercase and lowercase letters indicated significant differences of species richness and community diversity, respectively (Welch’s test, *P* < 0.05).

High-throughput sequencing analysis yielded a total of 1,970,283 raw tags from the 30 samples of various developmental stages of *G. molesta*. After quality filtering and removal of chimeric sequences, chloroplast, and mitochondrial sequences, 1,787,710 effective tags were obtained for subsequent analysis (Supplementary Table S1). The number of sequences per sample was normalized to 30,518 before analysis. A total of 24 bacterial phyla, 43 classes, 117 orders, 205 families, 402 genera, and 836 OTUs were identified across varying developmental stages of *G. molesta*. Rarefaction curves of sobs index at the OTU level reflected a saturated sampling depth (Supplementary Figure S1). Sequencing integrity was measured using Good’s coverage. The coverage of each sample was higher than 99%, suggesting that the majority of bacterial diversity in each sample had been captured in this study (Supplementary Table S2). Microbiota associated with eggs had high species richness with 189 OTUs identified at the initial stage. After hatching, bacterial species richness notably increased at first-instar larvae with 246 OTUs, then sharply decreased thereafter, and reached a minimum in the fifth-instar larvae. The number of OTUs in early-instar larvae (first and second instars) was significantly higher than that in late-instar larvae (third to fifth instars), showing a reduction in the microbial richness in the course of larval development. The number of OTUs increased again in male pupa and adults compared to late-instar larvae. The trend of species richness (Chao1) was similar to that of OTUs during the process of metamorphosis. However, the community diversity (Shannon) of adults was very low, and that of third-instar larvae was the lowest of all stages. Between sexes, bacterial species richness and diversity of male pupae were notably higher than those of female pupae, and there was no difference between adults (Figure 2 and Supplementary Table S2).

### Species Composition Across Different Developmental Stages of *G. molesta*

**FIGURE 3:**
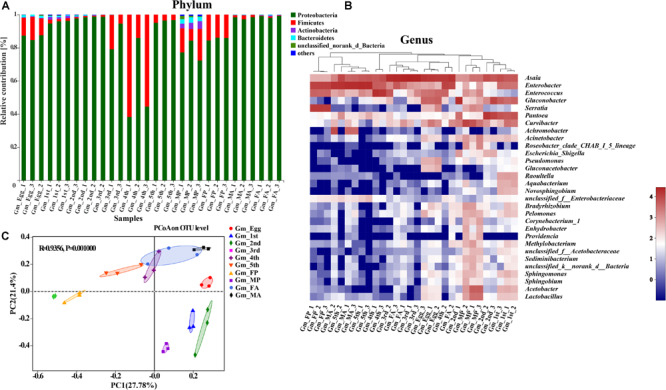
Gut bacterial dynamics across the development of *G. molesta.* **(A)** Relative abundance of bacterial communities at the phylum level in different treatments. **(B)** Heat map of major taxa at different life stages at the genus level generated by cluster analysis using the average method. Each column represents a single replicate for each of the 10 treatments. Columns were clustered according to the similarity of bacterial abundance profiles. Each row represents an OTU assigned to the genus level. Color gradients represent the abundance variation of different species in the sample. Plotting scale, from red to blue, indicates the decrease in relative abundance of bacteria. **(C)** Principal coordinate analysis (PCoA) of microbial communities according to host developmental stage (ANOSIM test, *P* = 0.001). Abbreviations: Gm = *Grapholita molesta*, 1st = first instar, 2nd = second instar, 3rd = third instar, 4th = fourth instar, 5th = fifth instar, FP = female pupa, MP = male pupa, FA = female adult, and MA = male adult.

The taxonomic analysis at phylum level revealed that the dominant phyla in the microbial community in relation to *G. molesta* were Proteobacteria (mean ± SE = 87.89% ± 2.67 of total sequences) and Firmicutes (10.07% ± 2.67), followed by Actinobacteria (0.63% ± 0.21), and Bacteroidetes (0.55% ± 0.16). There was an increasing trend from eggs to second-instar larvae in Proteobacteria abundance, which dramatically decreased from third-instar larvae and then went up again at the adult stage. Firmicutes abundance had the opposite trend, and both of them underwent notable changes during metamorphosis (Figure 3A). A few phyla that occurred at low abundance and sporadically in some samples are referred to as “others” (1% of the total sequences). At the genus level, a heat map was generated to visualize the distribution of multiple OTUs in different treatments. The top 30 abundant genera offered a detailed view of the bacterial community composition at different life stages (Figure 3B). The heat map also indicated a higher species richness and diversity in eggs and early-instar larvae than in other stages. Eggs shared a similar profile of bacterial types to third-instar larvae and female adults, whereas the gut microbiota of early-instar larvae appeared most similar to that of the male pupae. The common genera *Asaia*, *Enterococcus*, *Enterobacter*, *Pantoea*, *Gluconobacter*, *Acinetobacter*, *Pseudomonas*, *Lactobacillus*, *Curvibacter*, *Achromobacter*, and *Serratia* were found in the gut of *G. molesta*. *Asaia* was the most abundant genus (33.36%) compared to others and was present throughout metamorphosis. *Pantoea* (29.87%) and *Gluconobacter* (26.22%) were the dominant genera in early-instar larvae, followed by *Enterobacter* (5.63%), and *Curvibacter* (5.45%). These bacteria were also found in the eggs and are likely acquired from egg chorion during eclosion. However, *Enterobacter* (25.45%) and *Enterococcus* (18.91%) comprised major microbial components of late-instar larvae with a concomitant decrease in the abundance of *Pantoea* and *Gluconobacter*. After pupation, a significant rise in the abundance of *Serratia* (24.67%) was observed, especially in female pupae, with abundance very low at other stages. PCoA based on the Bray–Curtis distance showed relatively tight clustering according to developmental stages, and the dissimilarity of bacterial communities among different life stages was apparent (Figure 3C, ANOSIM, *R*^2^ = 0.9365, and *P* = 0.001). Therefore, bacterial community composition changed greatly with the growth and development of *G. molesta*.

**FIGURE 4:**
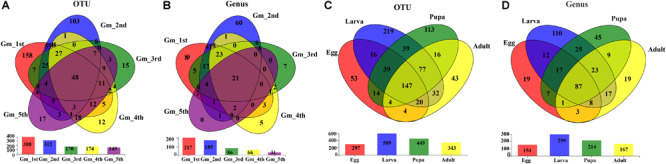
Venn diagrams depicting the overlap of the bacterial community at OTU and genus levels. **(A)** OTU level at the larval stage of *G. molesta*. **(B)** Genus level at the larval stage of *G. molesta*. **(C)** OTU level in the life cycle of *G. molesta*. **(D)** Genus level in the life cycle of *G. molesta*. Abbreviations: Gm = *Grapholita molesta*, 1st = first instar, 2nd = second instar, 3rd = third instar, 4th = fourth instar, 5th = fifth instar, FP = female pupa, MP = male pupa, FA = female adult, and MA = male adult.

The shared groups across the life cycle of *G. molesta* were shown in Venn diagrams (Figure 4). We found that 48 OTUs and 21 genera were shared among the larval stage (Figures 4A,B), of which 9 genera and 11 OTUs were abundant (Supplementary Table S3). A total of 147 OTUs and 87 genera were present throughout egg, larval, pupal, and adult stages (Figures 4C,D), of which six genera and nine OTUs were abundant (Supplementary Table S4). Their common genera and the corresponding OTUs were *Asaia* (OTU11), *Enterobacter* (OTU656), *Enterobacter* (OTU5), *Enterococcus* (OTU6), *Gluconobacter* (OTU272), *Pantoea* (OTU355), *Curvibacter* (OTU458), and *Enterobacter* (OTU15). OTU15 was abundant in late-instar larvae, and based on a comparison of its representative sequence with those available in GenBank, it was identified as *Enterobacter* (Supplementary Figure S2). The common presence of these genera suggests that they may have important functions in the process of growth and development of *G. molesta*.

### Significantly Different Bacterial Communities Across the Development of *G. molesta*

**FIGURE 5:**
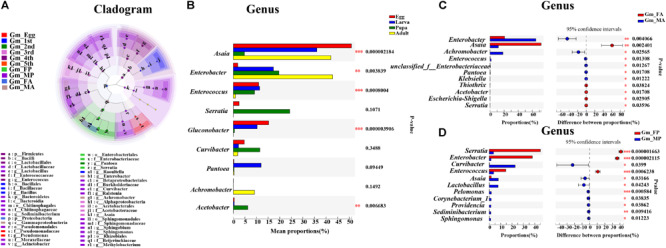
Significant difference analysis of the bacterial community in the development of *G. molesta*. **(A)** LEfSe analysis showing significant differences of microbial species at the level of phylum, class, order, family, and genus from inside to outside. Different color nodes represent microbiota that is significantly enriched at the corresponding life stages. Small yellow nodes indicate microbiota that has no significant difference at different life stages. **(B)** Significant differences of microbial composition in eggs, larvae, pupae, and adults (one-way ANOVA followed by Scheffe test, *P* ≤ 0.05). **(C)** Comparison of microbial species between FA and MA by independent *t* test. **(D)** Comparison of microbial species between FP and MP by independent *t* test. Abbreviations: Gm = *Grapholita molesta*, 1st = first instar, 2nd = second instar, 3rd = third instar, 4th = fourth instar, 5th = fifth instar, FP = female pupa, MP = male pupa, FA = female adult, and MA = male adult.

Linear discriminant analysis effect size analysis was performed to reveal the notable differences of gut bacteria from phylum to genus level across the life stages of *G. molesta* (Figure 5A). Each stage had its own significantly enriched set of microorganisms from phylum to genus. For example, at genus level, *Lactobacillus*, *Sediminibacterium*, *Sphingomonas*, *Methylobacterium*, *Ralstonia*, *Curvibacter*, and *Acinetobacter* were notably enriched in male pupae compared to other stages, whereas *Serratia* was the most abundant bacteria in female pupae. *Enterobacter* and *Achromobacter* were notably enriched in male adults. The top nine genera inhabiting in eggs, larvae, pupae, and adults also varied in abundance across the development of *G. molesta* (Figure 5B). Similar to LEfSe analysis, *Serratia* was abundant in pupae, and *Enterobacter* and *Achromobacter* were abundant in adults. Interestingly, sex-dependent bacterial communities were evident in both pupae and adults. The abundance of both *Enterobacter* and *Achromobacter* in male adults was significantly higher than that in female adults, whereas *Asaia* was enriched in female adults compared to males (Figure 5C). Similarly, significant differences in abundance of *Enterobacter*, *Curvibacter*, *Enterococcus, Asaia*, *Lactobacillus*, and *Sphingomonas* were also found between male and female pupae (Figure 5D).

### Microbial Interaction Networks in the Development of *G. molesta*

**FIGURE 6:**
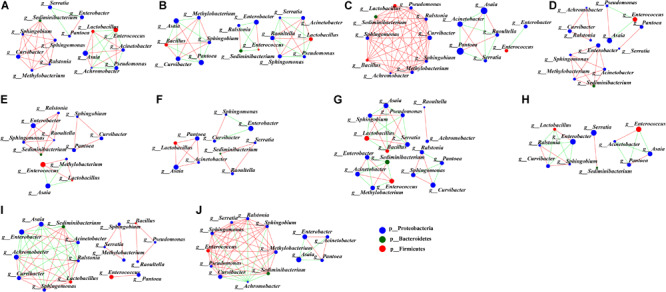
Network analysis applied to microbiota occurring in the development of *G. molesta*. Network analysis of gut microbiota in **(A)** eggs, **(B)** first-instar larvae, **(C)** second-instar larvae, **(D)** third-instar larvae, **(E)** fourth-instar larvae, **(F)** fifth-instar larvae, **(G)** male pupae, **(H)** female pupae, **(I)** male adults, and **(J)** female adults. The size of nodes represents the abundance of the genera. Node color corresponds to phylum taxonomic classification. Green edges represent co-exclusion/negative correlations, red edges represent co-occurrence/positive correlations between microbes, and the lines connecting each node represent the Spearman correlation coefficient values that were above 0.8 (red) or below -0.8 (green).

To uncover the co-occurrence pattern of bacteria at different life stages of *G. molesta*, a network was established based on significant correlations between different bacteria (Figure 6, Spearman’s *r* > 0.8, *P* < 0.01). In the network, the size of nodes represents the relative abundance of the genera. Green edges represent co-exclusion/negative correlations, and red edges represent co-occurrence/positive correlations between microbes. In the network of eggs (Figure 6A), the highly abundant bacteria *Enterococcus* and *Pseudomonas* had negative interactions with other bacteria, such as *Lactobacillus*, *Acinetobacter*, and *Achromobacter*. This may explain why the high abundance of *Enterococcus* and *Pseudomonas* was accompanied by low content of some other bacteria at the egg stage. At larval stage, we saw that interaction networks from early-instar larvae had more nodes and connections compared to networks from late-instar larvae. In the network of first-instar larvae (Figure 6B), the dominant genera *Asaia* and *Pantoea* form four mutually exclusive clusters with other bacteria, despite a positive correlation between them. In the network of second-instar larvae (Figure 6C), some moderately abundant bacteria such as *Curvibacter* and *Pseudomonas* had the most nodes and connections and co-occurred with most other bacteria. In the network of third- and fourth-instar larvae (Figures 6D,E), the highly abundant *Enterobacter* showed co-occurrence correlations with most bacteria, whereas the other highly abundant genus *Enterococcus* had little correlations with other bacteria. Unlike in third- and fourth-instar larvae, the dominant genus *Enterobacter* in fifth-instar larvae had negative connections with other bacteria (Figure 6F). The interaction network in male pupae (Figure 6G) was complicated compared to the network from female pupae (Figure 6H), and the abundant *Serratia* in male pupae had many correlations with other bacteria, while it only interacted with one bacterium (*Sediminibacterium*) in female pupae. This may explain the difference in abundance of *Serratia* between male pupae and female pupae. *Achromobacter* was abundant in male adults (Figure 6I) but relatively low in females, possibly due to its negative correlations with large numbers of bacteria in female adults (Figure 6J). These results showed that bacteria with high abundance did not necessarily have complex correlations with other bacteria. Conversely, bacteria with low or moderate abundance may play an important role in the microbial interaction network. The correlations of gut microbes at each developmental stage of *G. molesta* were different, which may explain the differences in the abundance of the same microbe at different life stages.

### Functional Prediction of Microbiota in the Development of *G. molesta*

**FIGURE 7:**
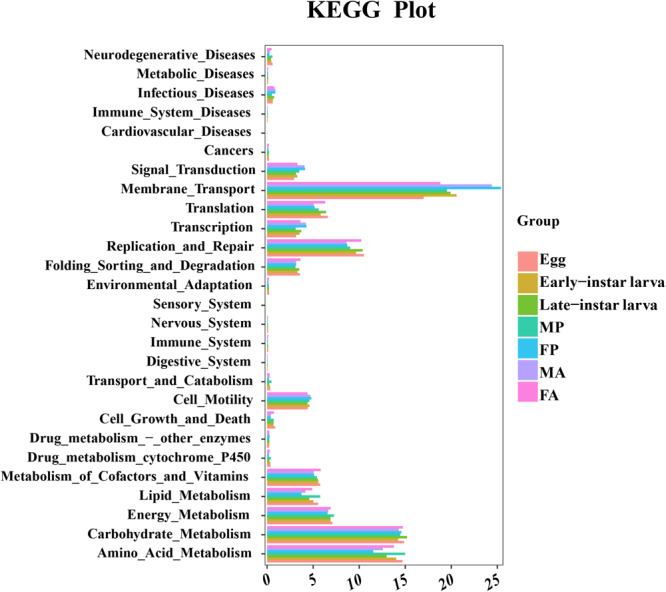
Comparison of predicted KEGG functions of gut bacteria in the development of *G. molesta*. Abbreviations: FP = female pupa, MP = male pupa, FA = female adult, and MA = male adult.

To better understand the important role of microbiota in *G. molesta*, the relative abundances of KEGG pathways were predicted by PICRUSt based on 16S rRNA gene sequences (Figure 7 and Supplementary Table S5). The functional categories, including genetic information processing (replication and repair), environmental information processing (membrane transport), and metabolism (carbohydrate and amino acid and energy metabolism), were enriched in all developmental stages of *G. molesta*. Despite the similarity of the gut microbiota metabolism and function across different life stages of *G. molesta*, changes in metabolic functions of microbiota could be observed. Notable differences between early-instar larvae and late-instar larvae were observed in the categories of amino acid, carbohydrate, and lipid metabolism and replication and repair. For different sexes, interestingly, amino acid, lipid, and energy metabolism were significantly enriched in males at pupal stage but enriched in females at the adult stage.

### Identification of Culturable Gut Bacteria in Fifth-Instar Larvae

Our culture-independent methods described above revealed a large number of microorganisms present in the midgut of *G. molesta*. Our culture-dependent results demonstrated that specific bacterial taxa resident within the midgut can grow *in vitro*. Analyses of 16S rRNA sequences of these cultivated bacteria revealed a total of 3 phyla, 12 genera, and 17 species (Table 1 and Supplementary Figure S3). Similar to the results of Illumina high-throughput sequencing, most of the bacteria isolated from cultures belonged to Proteobacteria, Firmicutes, and Actinobacteria.

**TABLE 1:** NCBI BLAST results of the 16S rRNA gene sequences from midgut bacteria isolates.

**Sequence**	**Identification**	**GenBank Accession No.**	**Similar Sequence/Accession No.**	**Identity %**	**Phylum**
Gm1	*Bacillus altitudinis*	MN826158	*Bacillus altitudinis*/MN710447.1	100	Firmicutes
Gm2	*Bacillus cereus*	MN826159	*Bacillus cereus*/MH762120.1	100	Firmicutes
Gm3	*Enterococcus mundtii*	MN826160	*Enterococcus mundtii*/MK414812.1	100	Firmicutes
Gm4	*Enterococcus casseliflavus*	MN826161	*Enterococcus casseliflavus*/KJ803876.1	99.65	Firmicutes
Gm5	*Staphylococcus epidermidis*	MN826162	*Staphylococcus epidermidis*/LR735440.1	100	Firmicutes
Gm6	*Planococcus* sp.	MN826163	*Planococcus maritimus*/KR063196.1	100	Firmicutes
Gm7	*Staphylococcus* sp.	MN826164	*Staphylococcus warneri*/MN181250.1	100	Firmicutes
Gm8	*Gordonia* sp.	MN826165	*Gordonia terrae/*MN103754.1	100	Actinobacteria
Gm9	*Kocuria rosea*	MN826166	*Kocuria rosea*/MG190715.1	99.93	Actinobacteria
Gm10	*Curtobacterium flaccumfaciens*	MN826167	*Curtobacterium flaccumfaciens*/MK398100.1	100	Actinobacteria
Gm11	*Microbacterium esteraromaticum*	MN826168	*Microbacterium esteraromaticum*/MG719571.1	100	Actinobacteria
Gm12	*Enterobacter ludwigii*	MN826169	*Enterobacter ludwigii*/MG836087.1	100	Proteobacteria
Gm13	*Enterobacter cancerogenus*	MN826170	*Enterobacter cancerogenus*/CP025225.1	99.86	Proteobacteria
Gm14	*Sphingomonas* sp.	MN826171	*Sphingomonas* sp./MK612129.1	99.85	Proteobacteria
Gm15	*Brevundimonas aurantiaca*	MN826172	*Brevundimonas aurantiaca*/MN187256.1	99.92	Proteobacteria
Gm16	*Erwinia persicina*	MN826173	*Erwinia persicina*/HQ220163.1	98.73	Proteobacteria
Gm17	*Erwinia toletana*	MN826174	*Erwinia toletana*/JX134630.1	99.28	Proteobacteria

## Discussion

Bacteria, as commensals, mutualists, parasites, and pathogens, play important roles in shaping the ecology and evolution of their hosts (Moran, 2007; Noda et al., 2007). Gut bacteria are actively involved in insect host physiology, behavior, and ecology (Oliver et al., 2003; Dillon and Dillon, 2004; Freitak et al., 2009; Sharon et al., 2010; Hosokawa and Fukatsu, 2020). Interactions between gut bacteria and insect hosts are complex, and most studies have focused on larval gut bacteria (Visôtto et al., 2009; Tang et al., 2012). In the present work, the microbial community composition of *G. molesta* across the entire life cycle was investigated. Alpha diversity of gut microbiota varied across the life stages of *G. molesta*. Species richness in early-instar larvae was greater than that in late-instar larvae. This was opposite to *B. mori* (Chen et al., 2018); species richness in first-instar larvae of *B. mori* was the lowest and increased thereafter and reached a maximum in the fifth-instar larvae. Results for *G. molesta* were more similar to those for *S. littoralis* and the natural enemy insect *C. sinica* (Chen et al., 2016; Zhao et al., 2019), whose bacterial richness in early-instar larvae was higher than that in late-instar larvae. This may suggest that the host’s physiology affects the microbiota composition or interactions. Considering that the larval stage of Lepidoptera ingests a large number of plant materials and fruits, the microbes that are highly abundant in early-instar larvae may enable the host to better adjust to various environments, such as metabolizing insecticides or interfering with potentially pathogenic microbes present in its food. A previous work found that Proteobacteria, followed by Firmicutes, were the major phyla present in the gut of *G. molesta* larvae feeding on fruits or shoots (Liu et al., 2019). Our study showed that Proteobacteria and Firmicutes were the dominant phyla at each life stage of *G. molesta*. Members of Proteobacteria have been shown to have the ability to provide nutrients for host insects at their early life stages by degrading major structural components of plant materials, e.g., *Pantoea* spp. in the leaf-cutter ant *Atta colombica* (Suen et al., 2010). Members of Firmicutes, such as *Clostridia*, have been shown to harvest energy from their diet by degrading cellulose and hemicellulose and to metabolize amino acids (Fonknechten et al., 2010). The highly abundant Proteobacteria and Firmicutes observed in our research suggests that they may be important in nutrient absorption and energy metabolism.

Shifts of gut microbial community from *Gluconobacter* and *Pantoea* in early-instar larvae to *Enterococcus* and *Enterobacter* in late-instar larvae and to *Serratia* in pupae were apparent, in concert with the changes of host developmental processes. These particular microbial taxa may help the host utilize life-stage-specific resources by providing functions related to digestion, detoxification, and nutrient supplementation. For example, *Pantoea* in desert locust *S. gregaria* is putatively helpful in defense against pathogens (Dillon and Charnley, 1995). *Gluconobacter* could be involved in host metabolism by supplying nutrients or oxidizing certain substrates or contributing to the maintenance of host gut homeostasis (Ryu et al., 2008). The high abundance of *Gluconobacter* and *Pantoea* in early-instar larvae of *G. molesta* may help them fight pathogens and obtain nutrients. *Enterococcus* has been found to be one of the dominant gut microorganisms and presents at high frequency in other Lepidoptera insects, such as *Cydia pomonella* (Lepidoptera: Tortricidae; Liu et al., 2019), *Lymantria dispar* (Lepidoptera: Lymantriidae; Broderick et al., 2004), *Helicoverpa armigera* (Lepidoptera: Noctuidae; Xiang et al., 2006), and *S. littoralis* (Chen et al., 2016; Shao et al., 2017), suggesting that *Enterococcus* may perform some conserved functions in these highly phytophagous insects. For example, a stable isotope labeling-based approach indicates high metabolic activity of *Enterococcus mundtii* inside its host *S. littoralis* (Shao et al., 2014). *E. mundtii* in the midgut of *S. littoralis* can also prevent colonization of pathogens by secreting a stable antimicrobial peptide (Shao et al., 2017). *Enterococcus* in gypsy moth *L. dispar* and spruce budworm *Choristoneura fumiferana* (Lepidoptera: Tortricidae) protects their hosts from *Bacillus thuringiensis* and grows normally in chlorpyrifos ethyl, lambda-cyhalothrin, spinosad, and lufenuron selective medium (van Frankenhuyzen et al., 2010; Almeida et al., 2017), suggesting that they play a role in the tolerance to toxic substances. Liu et al. (2019) found low abundance of *Enterococcus* in the gut of fifth-instar larvae of fruit-feeding *G. molesta* but higher abundance in shoot-feeding *G. molesta*, which was not consistent with our findings. We speculate that this may due to differences in diet or habitat. Future research with the cultivable *E. mundtii* and *Enterococcus casseliflavus* in *G. molesta* might shed light on their functions. *Serratia* has been proven to play several roles in defending hosts against parasitoids (Costopoulos et al., 2014). *Serratia marcescens* is reported as an entomopathogenic bacterium that opportunistically infects a wide range of hosts, from honey bees (El Sanousi et al., 1987; Raymann et al., 2017) to humans (Kurz et al., 2003; Nehme et al., 2007; Stathopoulos et al., 2014). A large number of *S. marcescens* were also detected in pupae of *G. molesta*. However, the function of *S. marcescens* in pupae is unclear. Virulence of *S. marcescens* may depend on unusual conditions under which it becomes abundant in the gut. Therefore, further research is needed to verify whether *S. marcescens* may be an opportunistic pathogen to *G. molesta* larvae by oral exposure experiments. A previous study has shown that host diet affects diversity and composition of gut microbiota in *G. molesta* (Liu et al., 2019). Our results showed that gut microbiota composition and diversity in *G. molesta* can also be significantly affected by host developmental stage. Such shifts can be very important for insects to overcome plant defenses and adjust to complex digestive environments.

Sex-dependent bacterial community differences in the gut of *G. molesta* were detected in this study. The bacterial community in female pupae was not as diverse as that in males. This was similar to black flies (Diptera: Simuliidae), whose bacterial composition differed between males and females from the same habitat (Tang et al., 2012). It is possible that females are more immune-competent than males in response to invasion and colonization by microorganisms (Kurtz et al., 2000). Alternatively, these disparities may reflect sex-specific functional metabolic differences. For example, amino acid, lipid, and energy metabolism were significantly enhanced in males at the pupal stage but enhanced in females at the adult stage. Higher levels of metabolism in male pupae may be related to preparation for mating, while those in female adults may be related to preparation for oviposition. This is the first report of a sex-dependent difference in gut bacterial communities in *G. molesta* pupae and adults.

Microbial interactions occurring within different life stages of *G. molesta* may influence symbiont colonization, which likely affects bacterial diversity. For example, *Serratia* isolated from *Aedes albopictus* (Diptera: Culicidae) is unable to effectively infect *Aedes aegypti* (Diptera: Culicidae) when it possesses its native microbiome but achieves a higher titer when applied to axenic larvae (Coon et al., 2014). Both *E. faecalis* and *E. casseliflavus* are ubiquitous in the environment and often present in high densities in the gut of early-instar *S. littoralis* larvae prior to the establishment of *E. mundtii*, because *E. mundtii* secretes a stable antimicrobial peptide that prevents colonization by *E. faecalis* and *E. casseliflavus* (Fonknechten et al., 2010; Shao et al., 2017). In this study, microbial interaction networks were analyzed to provide information on potential interaction patterns of microbes across the life cycle of *G. molesta*. The more edges, the more connected the species is to other species. There may be no specific relationship between the number of edges and the abundance of microbes. For example, the dominant genus *Asaia* correlated with the majority of bacteria at some stages of *G. molesta* development, but not at all stages, even though it was abundant at all life stages. Conversely, some bacteria of low or moderate abundance may still play an important role in microbial interactions. The correlations of intestinal microbes differed among developmental stages of *G. molesta*, and the various positive and negative correlations may result in the different abundance of the particular bacteria across life stages. For example, *Achromobacter* was abundant in male adults but extremely low in females, which may be due to its negative correlations with large numbers of other bacteria in female adults, such as *Sphingomonas* and *Ralstonia*. Microbial interactions at each life stage of *G. molesta* appear complex, and further work is required to determine the functions of these multi-interacting partners and how the positive and negative interactions among bacteria come about. Artificial manipulations of the microbial composition of *G. molesta* may be particularly useful in this regard.

Despite variation in bacterial communities at different developmental stages of *G. molesta*, a similar functional profile analyzed by PICRUSt was observed. The microbial communities within the gut of *G. molesta* can perform many metabolic functions. As reported by Liu et al. (2019), carbohydrate and amino acid metabolism and energy metabolism were enriched in the fifth-instar larvae of *G. molesta*. Our results showed that they were enriched at all life stages of *G. molesta*, likely because these functions are essential to the survival of all life stages. PICRUSt provides little information about the functions of gut microbiota in host physiology, so metagenomic and metatranscriptomic approaches will be essential to illustrate the microbial functions in future work. However, the importance of traditional culture-dependent methods should not be ignored. The colonies obtained from *G. molesta* by traditional methods should contribute to subsequent verification of their functions in terms of pathogenicity and host fitness, especially the bacteria from the genus *Enterococcus*.

In summary, this is the first description of the overall structure of gut microbiota across the life stages of *G. molesta*. A major limitation of this work is that all data generated derive from a single generation of a single laboratory population; far greater complexity might be expected in wild populations, given that most bacterial colonization depends on environmental exposure. Our results showed that gut microbiota of the *G. molesta* varied greatly among developmental stages and between sexes. Microbial interactions across the life cycle were complex, and their corresponding functions were unclear. 16S rRNA sequencing provides little information about metabolic capabilities of bacteria. Combining studies of 16S rRNA with metagenomic analysis may be useful in this regard. Altogether, an improved understanding of gut microbiota dynamics across the life cycle of *G. molesta* provides the basis for elucidating the metabolic functions of gut microbiota and contributes to the development of novel biocontrol strategies against fruit-boring pests within the Lepidoptera.

## Data Availability Statement

The raw data obtained by Illumina high-throughput sequencing technology in this study were deposited in the NCBI short-read archive (SRA) under accession number SRP256116. The nucleotide sequences of isolates 1–17 were submitted to the GenBank database with accession number [GenBank: MN826158–MN826174].

## Author Contributions

XW performed the experiments and wrote the first draft of the manuscript. SS, XY, JC, and HW helped with the analysis of the results. ZL and JM revised the manuscript and clarified the English composition. XL conceived of and designed the study and edited the manuscript. All authors contributed to the article and approved the submitted version.

## Conflict of Interest

The authors declare that the research was conducted in the absence of any commercial or financial relationships that could be construed as a potential conflict of interest.
