# Supplementary material for: Variability of Gut Microbiota Across the Life Cycle of Grapholita molesta (Lepidoptera: Tortricidae)
Source: Front Microbiol. 2020 Jun 30;11:1366. doi: 10.3389/fmicb.2020.01366 (PMC7340173; doi:10.3389/fmicb.2020.01366)
Supplement: Supplementary file 1 [file Data_Sheet_1.docx]

**FIGURE S1|** Rarefaction curves of sobs index reﬂected a saturated sampling depth. Abbreviations: Gm: *Grapholita molesta*, 1st = first instar, 2nd = second instar, 3rd = third instar, 4th = fourth instar, 5th = fifth instar, FP = female pupa, MP = male pupa, FA = female adult, MA = male adult.

**FIGURE S2|** Maximum Likelihood (Tamura-Nei model) phylogenetic analysis of OTU15 identified from gut microbiota of *G. molesta.* Bootstrap values are shown in the branches based on 1000 replicates. OTU656 and OTU5 belonged to the genus of *Enterobacter* were the sequences obtained in the study.

**FIGURE S****3 |** Neighbor-joining phylogenetic tree (p-distance method) of bacterial isolates obtained from the midgut of *G. molesta*. Bootstrap values are shown in the branches based on 1000 replicates. Abbreviations: Gm: *Grapholita molesta.*

| Table S1**\|** Tags analysis of all samples from different life stages of *G. molesta* | | | |
| --- | --- | --- | --- |
| Sample ID | Raw Tags | Clean Tags | Effective Tags |
| Gm_Egg_1 | 45554 | 43180 | 41791 |
| Gm_Egg_2 | 58381 | 55964 | 53819 |
| Gm_Egg_3 | 89952 | 83286 | 80258 |
| Gm_1st_1 | 35996 | 33041 | 31021 |
| Gm_1st_2 | 55710 | 51065 | 48545 |
| Gm_1st_3 | 58508 | 54130 | 50674 |
| Gm_2nd_1 | 56516 | 53367 | 51636 |
| Gm_2nd_2 | 76830 | 71694 | 69632 |
| Gm_2nd_3 | 45093 | 42584 | 41427 |
| Gm_3rd_1 | 54471 | 52343 | 51861 |
| Gm_3rd_2 | 74691 | 71146 | 70546 |
| Gm_3rd_3 | 62664 | 59644 | 58837 |
| Gm_4th_1 | 60517 | 56270 | 55455 |
| Gm_4th_2 | 78696 | 73171 | 71550 |
| Gm_4th_3 | 116040 | 107414 | 105635 |
| Gm_5th_1 | 62697 | 57778 | 55857 |
| Gm_5th_2 | 65558 | 61250 | 59457 |
| Gm_5th_3 | 47592 | 43165 | 39113 |
| Gm_FP_1 | 64255 | 59222 | 58422 |
| Gm_FP_2 | 75955 | 69625 | 68386 |
| Gm_FP_3 | 115495 | 106542 | 104823 |
| Gm_MP_1 | 36656 | 31476 | 30518 |
| Gm_MP_2 | 47705 | 41974 | 40501 |
| Gm_MP_3 | 56554 | 53254 | 50560 |
| Gm_FA_1 | 60081 | 57469 | 56971 |
| Gm_FA_2 | 72191 | 67788 | 66857 |
| Gm_FA_3 | 59678 | 57278 | 56553 |
| Gm_MA_1 | 62168 | 58419 | 57426 |
| Gm_MA_2 | 77537 | 72508 | 71660 |
| Gm_MA_3 | 96542 | 89673 | 87919 |
| Total | 1970283 | 1835720 | 1787710 |

Table S2**|** Alpha diversity index in the structure of microbiota associated with different life stages of *G. molesta*

| Sample ID | Number of OTUs | Chao1 | Shannon | Coverage |
| --- | --- | --- | --- | --- |
| Gm_Egg_1 | 195 | 229.44 | 2.08834 | 0.99862 |
| Gm_Egg_2 | 172 | 205.056 | 2.0659 | 0.99885 |
| Gm_Egg_3 | 181 | 206.87 | 1.96941 | 0.99885 |
| Gm_1st_1 | 222 | 263.625 | 3.12118 | 0.99879 |
| Gm_1st_2 | 229 | 262.476 | 2.98158 | 0.99876 |
| Gm_1st_3 | 251 | 266.75 | 2.77126 | 0.99908 |
| Gm_2nd_1 | 198 | 218.813 | 1.59041 | 0.99879 |
| Gm_2nd_2 | 187 | 222.455 | 1.57983 | 0.99869 |
| Gm_2nd_3 | 175 | 230.682 | 1.59741 | 0.99836 |
| Gm_3rd_1 | 110 | 147.188 | 0.50041 | 0.99885 |
| Gm_3rd_2 | 122 | 165.333 | 1.05854 | 0.99869 |
| Gm_3rd_3 | 74 | 111.8 | 0.49743 | 0.99908 |
| Gm_4th_1 | 115 | 173.333 | 1.44804 | 0.99836 |
| Gm_4th_2 | 107 | 132.5 | 1.67219 | 0.99889 |
| Gm_4th_3 | 103 | 133 | 1.47686 | 0.99882 |
| Gm_5th_1 | 96 | 159.909 | 1.78562 | 0.99876 |
| Gm_5th_2 | 108 | 147.375 | 1.83554 | 0.99882 |
| Gm_5th_3 | 107 | 126.462 | 1.91271 | 0.99925 |
| Gm_FP_1 | 115 | 164.5 | 1.38687 | 0.99853 |
| Gm_FP_2 | 110 | 174.5 | 1.39232 | 0.99859 |
| Gm_FP_3 | 116 | 133.714 | 1.39556 | 0.99895 |
| Gm_MP_1 | 158 | 191.333 | 2.97564 | 0.99918 |
| Gm_MP_2 | 194 | 217.214 | 3.32097 | 0.99915 |
| Gm_MP_3 | 254 | 283.063 | 3.48357 | 0.99898 |
| Gm_FA_1 | 143 | 163.308 | 0.78425 | 0.99892 |
| Gm_FA_2 | 183 | 210.333 | 1.37619 | 0.99866 |
| Gm_FA_3 | 138 | 163.87 | 0.80004 | 0.99885 |
| Gm_MA_1 | 152 | 199.04 | 1.25548 | 0.99839 |
| Gm_MA_2 | 165 | 247.65 | 1.35489 | 0.9981 |
| Gm_MA_3 | 137 | 201.474 | 1.26249 | 0.99836 |

| **TABLE S3\|** Dominant genera and corresponding OTUs across the life cycle of *G. molesta* | | | |
| --- | --- | --- | --- |
| **Phylum** | **Family** | **Genus** | **OTU** |
| p__Proteobacteria | f__Acetobacteraceae | *g__Asaia* | OTU11 |
| p__Proteobacteria | f__Enterobacteriaceae | *g__Enterobacter* | OTU656 |
| p__Firmicutes | f__Enterococcaceae | *g__Enterococcus* | OTU6 |
| p__Proteobacteria | f__Acetobacteraceae | *g__Gluconobacter* | OTU272 |
| p__Proteobacteria | f__Enterobacteriaceae | *g__Serratia* | OTU585 |
| p__Proteobacteria | f__Enterobacteriaceae | *g__Pantoea* | OTU355 |
| p__Proteobacteria | f__Burkholderiaceae | *g__Curvibacter* | OTU458 |
| p__Proteobacteria | f__Enterobacteriaceae | *g__Enterobacter* | OTU5 |
| p__Proteobacteria | f__Enterobacteriaceae | *g_ Enterobacter* | OTU15 |
| p__Proteobacteria | f__Burkholderiaceae | *g__Achromobacter* | OTU651 |
| p__Proteobacteria | f__Acetobacteraceae | *g__Acetobacter* | OTU567 |

| **TABLE S4\|** Dominant genera and corresponding OTUs at the larval stage of *G. molesta* | | | |
| --- | --- | --- | --- |
| **Phylum** | **Family** | **Genus** | **OTU** |
| p__Proteobacteria | f__Acetobacteraceae | *g__Asaia* | OTU11 |
| p__Firmicutes | f__Enterococcaceae | *g__Enterococcus* | OTU6 |
| p__Proteobacteria | f__Acetobacteraceae | *g__Gluconobacter* | OTU272 |
| p__Proteobacteria | f__Enterobacteriaceae | *g__Pantoea* | OTU355 |
| p__Proteobacteria | f__Enterobacteriaceae | *g__Enterobacter* | OTU656 |
| p__Proteobacteria | f__Enterobacteriaceae | *g__Enterobacter* | OTU5 |
| p__Proteobacteria | f__Enterobacteriaceae | *g__ Enterobacter* | OTU15 |
| p__Proteobacteria | f__Enterobacteriaceae | *g__Pantoea* | OTU226 |
| p__Proteobacteria | f__Burkholderiaceae | *g__Curvibacter* | OTU458 |

**TABLE S5|** Abundance of KEGG functional prediction (%)

|  | **Egg1** | **Egg2** | **Egg3** | **Early-instar larva1** | **Early-instar larvav2** | **Late-instar larva1** | **Late-instar larva2** | **Late-instar larva3** | **MP1** | **MP2** | **MP3** | **FP1** | **FP2** | **FP3** | **MA1** | **MA2** | **MA3** | **FA1** | **FA2** | **FA3** |
| --- | --- | --- | --- | --- | --- | --- | --- | --- | --- | --- | --- | --- | --- | --- | --- | --- | --- | --- | --- | --- |
| **Amino Acid Metabolism** | **14.80** | **14.52** | **14.75** | **13.90** | **14.13** | **14.91** | **12.06** | **11.96** | **14.99** | **15.16** | **14.79** | **11.51** | **11.52** | **11.52** | **12.44** | **12.88** | **12.39** | **14.59** | **13.12** | **13.59** |
| **Carbohydrate Metabolism** | **14.72** | **14.88** | **14.98** | **14.33** | **14.13** | **15.27** | **15.85** | **14.47** | **14.54** | **14.09** | **14.74** | **14.60** | **14.58** | **14.58** | **14.36** | **14.13** | **14.39** | **14.79** | **14.63** | **14.78** |
| **Energy Metabolism** | **7.04** | **7.04** | **7.19** | **7.03** | **6.73** | **7.11** | **6.77** | **6.77** | **7.21** | **7.09** | **7.46** | **6.58** | **6.58** | **6.58** | **6.64** | **6.63** | **6.64** | **7.01** | **6.79** | **6.89** |
| **Lipid Metabolism** | **5.67** | **5.46** | **5.49** | **4.99** | **5.05** | **5.54** | **4.15** | **4.03** | **5.91** | **6.01** | **5.36** | **3.73** | **3.73** | **3.73** | **4.10** | **4.31** | **4.07** | **5.38** | **4.56** | **4.75** |
| **Metabolism of Cofactors and Vitamins** | **5.64** | **5.69** | **5.95** | **5.60** | **5.51** | **6.23** | **4.86** | **5.49** | **5.34** | **5.19** | **5.68** | **5.11** | **5.12** | **5.12** | **5.11** | **4.94** | **5.13** | **6.05** | **5.52** | **5.87** |
| **Drug metabolism - cytochrome P450** | **0.39** | **0.37** | **0.36** | **0.34** | **0.35** | **0.34** | **0.21** | **0.20** | **0.44** | **0.43** | **0.36** | **0.20** | **0.19** | **0.19** | **0.23** | **0.25** | **0.22** | **0.34** | **0.27** | **0.27** |
| **Drug metabolism - other enzymes** | **0.22** | **0.22** | **0.22** | **0.25** | **0.24** | **0.21** | **0.27** | **0.28** | **0.22** | **0.22** | **0.27** | **0.28** | **0.28** | **0.28** | **0.27** | **0.26** | **0.27** | **0.22** | **0.25** | **0.24** |
| **Cell Growth and Death** | **0.86** | **0.88** | **0.93** | **0.68** | **0.71** | **1.03** | **0.60** | **0.48** | **0.75** | **0.69** | **0.73** | **0.39** | **0.39** | **0.39** | **0.41** | **0.39** | **0.41** | **0.88** | **0.60** | **0.73** |
| **Cell Motility** | **4.58** | **4.45** | **4.20** | **4.95** | **4.25** | **3.93** | **4.32** | **4.90** | **4.58** | **5.23** | **3.94** | **4.82** | **4.81** | **4.81** | **4.70** | **4.71** | **4.68** | **4.30** | **4.58** | **4.31** |
| **Transport and Catabolism** | **0.38** | **0.33** | **0.32** | **0.34** | **0.29** | **0.26** | **0.22** | **0.23** | **0.49** | **0.53** | **0.41** | **0.21** | **0.21** | **0.21** | **0.27** | **0.31** | **0.26** | **0.29** | **0.27** | **0.24** |
| **Digestive System** | **0.06** | **0.06** | **0.06** | **0.05** | **0.05** | **0.06** | **0.07** | **0.05** | **0.04** | **0.04** | **0.05** | **0.05** | **0.05** | **0.05** | **0.06** | **0.05** | **0.06** | **0.06** | **0.06** | **0.06** |
| **Immune System** | **0.11** | **0.12** | **0.13** | **0.10** | **0.12** | **0.16** | **0.08** | **0.10** | **0.07** | **0.07** | **0.08** | **0.08** | **0.08** | **0.08** | **0.09** | **0.09** | **0.09** | **0.14** | **0.11** | **0.13** |
| **Nervous System** | **0.10** | **0.10** | **0.11** | **0.09** | **0.11** | **0.11** | **0.07** | **0.07** | **0.08** | **0.09** | **0.10** | **0.05** | **0.06** | **0.06** | **0.07** | **0.07** | **0.07** | **0.10** | **0.08** | **0.09** |
| **Sensory System** | **0.00** | **0.00** | **0.00** | **0.00** | **0.00** | **0.00** | **0.00** | **0.00** | **0.00** | **0.00** | **0.00** | **0.00** | **0.00** | **0.00** | **0.00** | **0.00** | **0.00** | **0.00** | **0.00** | **0.00** |
| **Environmental Adaptation** | **0.22** | **0.22** | **0.23** | **0.20** | **0.20** | **0.24** | **0.21** | **0.18** | **0.19** | **0.19** | **0.19** | **0.16** | **0.16** | **0.16** | **0.16** | **0.16** | **0.16** | **0.22** | **0.18** | **0.20** |
| **Folding, Sorting and Degradation** | **3.46** | **3.52** | **3.71** | **3.29** | **3.33** | **3.99** | **3.15** | **3.34** | **3.04** | **2.94** | **3.27** | **3.15** | **3.15** | **3.15** | **3.18** | **3.08** | **3.19** | **3.78** | **3.41** | **3.68** |
| **Replication and Repair** | **10.17** | **10.41** | **10.92** | **9.58** | **9.75** | **11.72** | **9.99** | **9.38** | **8.87** | **8.54** | **9.65** | **8.71** | **8.70** | **8.70** | **8.71** | **8.44** | **8.74** | **10.78** | **9.53** | **10.35** |
| **Transcription** | **3.12** | **3.18** | **3.15** | **3.54** | **3.50** | **3.04** | **3.97** | **4.14** | **3.04** | **3.02** | **3.25** | **4.27** | **4.27** | **4.28** | **4.25** | **4.14** | **4.26** | **3.26** | **3.88** | **3.69** |
| **Translation** | **6.36** | **6.54** | **6.87** | **5.72** | **5.91** | **7.52** | **6.20** | **5.47** | **5.52** | **5.22** | **6.05** | **5.17** | **5.16** | **5.16** | **5.11** | **4.95** | **5.13** | **6.77** | **5.76** | **6.38** |
| **Membrane Transport** | **17.49** | **17.49** | **16.01** | **19.92** | **21.21** | **14.27** | **22.43** | **23.04** | **19.69** | **20.08** | **18.82** | **25.40** | **25.39** | **25.38** | **24.26** | **24.67** | **24.24** | **16.44** | **21.15** | **18.81** |
| **Signal Transduction** | **3.06** | **2.94** | **2.77** | **3.54** | **3.04** | **2.38** | **3.17** | **3.86** | **3.50** | **3.77** | **3.22** | **4.11** | **4.12** | **4.12** | **4.06** | **4.05** | **4.06** | **2.94** | **3.66** | **3.27** |
| **Cancers** | **0.21** | **0.21** | **0.21** | **0.20** | **0.19** | **0.22** | **0.12** | **0.17** | **0.22** | **0.20** | **0.22** | **0.15** | **0.15** | **0.15** | **0.16** | **0.16** | **0.16** | **0.22** | **0.18** | **0.20** |
| **Cardiovascular Diseases** | **0.01** | **0.01** | **0.01** | **0.01** | **0.00** | **0.00** | **0.00** | **0.00** | **0.02** | **0.02** | **0.02** | **0.00** | **0.00** | **0.00** | **0.00** | **0.00** | **0.00** | **0.00** | **0.00** | **0.00** |
| **Immune System Diseases** | **0.06** | **0.07** | **0.07** | **0.06** | **0.06** | **0.07** | **0.07** | **0.06** | **0.07** | **0.06** | **0.08** | **0.06** | **0.06** | **0.06** | **0.06** | **0.06** | **0.06** | **0.06** | **0.06** | **0.06** |
| **Infectious Diseases** | **0.58** | **0.60** | **0.63** | **0.69** | **0.57** | **0.65** | **0.74** | **0.92** | **0.49** | **0.48** | **0.58** | **0.89** | **0.90** | **0.90** | **0.91** | **0.86** | **0.92** | **0.70** | **0.83** | **0.82** |
| **Metabolic Diseases** | **0.10** | **0.11** | **0.11** | **0.10** | **0.10** | **0.12** | **0.12** | **0.10** | **0.09** | **0.08** | **0.10** | **0.10** | **0.10** | **0.10** | **0.09** | **0.09** | **0.09** | **0.11** | **0.10** | **0.11** |
| **Neurodegenerative Diseases** | **0.59** | **0.58** | **0.61** | **0.48** | **0.46** | **0.64** | **0.32** | **0.31** | **0.59** | **0.55** | **0.59** | **0.23** | **0.23** | **0.23** | **0.30** | **0.32** | **0.30** | **0.58** | **0.40** | **0.47** |
