# Supplementary figures and images for: Variability of Gut Microbiota Across the Life Cycle of Grapholita molesta (Lepidoptera: Tortricidae)
Source: Front Microbiol. 2020 Jun 30;11:1366. doi: 10.3389/fmicb.2020.01366 (PMC7340173; doi:10.3389/fmicb.2020.01366)

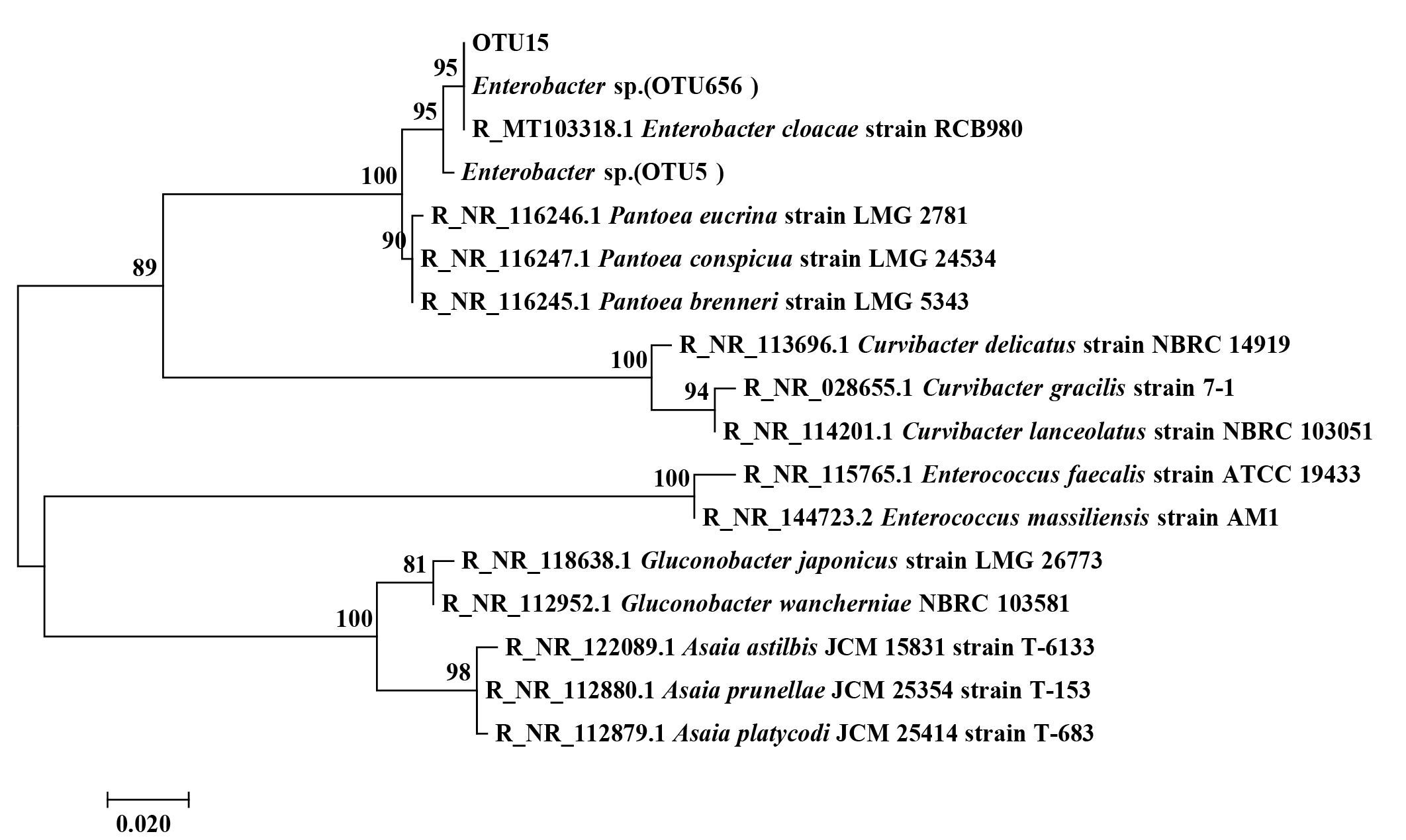

Supplement: Supplementary file 3 [file Image_2.tif]

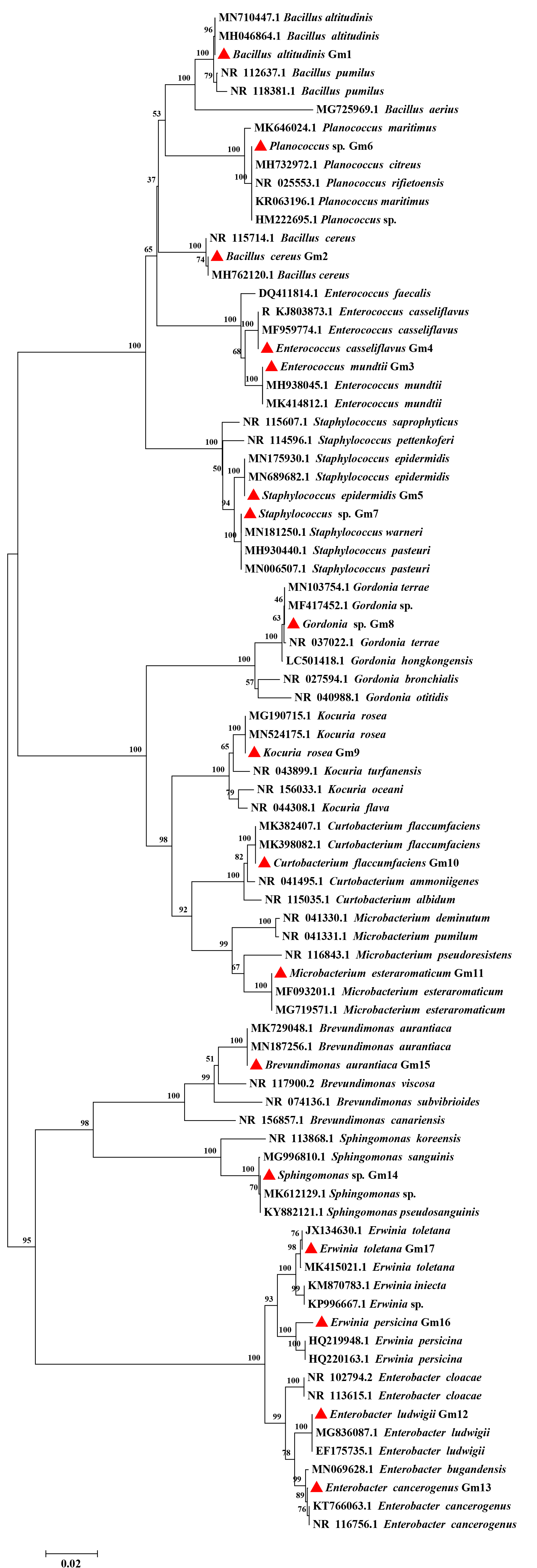

Supplement: Supplementary file 4 [file Image_3.tif]
